# Editorial: Calcium homeostasis in skeletal muscle function, plasticity and disease, Volume II

**DOI:** 10.3389/fphys.2023.1130073

**Published:** 2023-01-20

**Authors:** Matias Mosqueira, Heinrich Brinkmeier, Enrique Jaimovich

**Affiliations:** ^1^ Institute of Physiology and Pathophysiology, Heidelberg University Hospital, Heidelberg, Germany; ^2^ Institute of Pathophysiology University Medicine Greifswald, Greifswald, Germany; ^3^ Center for Exercise, Metabolism, and Cancer Studies, Faculty of Medicine, University of Chile, Santiago, Chile

**Keywords:** excitation-contraction coupling, SOCE, calcium entry unit, dysferlin, casequestrin-1, skeletal muscle tissue engineering, C2C12, nitric oxide

Since 1947, when Lewis Victor Heilbrunn described the fundamental role of calcium as the only ion able to produce muscle contraction, the understanding of the phenomenon has been expanded to explain its intracellular signaling activity, regulation of the metabolism, tissue formation, development, and regeneration. This protagonist of the physiological and biochemical processes of skeletal muscle was and is fundamental to comprehending distinct pathologies turning it into a key target for therapeutic strategies. The second Research Topic *Calcium homeostasis in skeletal muscle function, plasticity and disease* collected new and relevant information on the role of calcium in skeletal muscle, contributing to the progress of establishing a new point of muscle research. For instance, Bolaños and Calderón extensively and critically reviewed the established knowledge of the complex process of excitation-contraction coupling (ECC), bringing new processes and actors that facilitated the comprehension of muscle contraction. It is interesting to note that based on the historical pieces of evidence of ECC elucidation, they put together the development of new fast and low-affinity calcium dyes to describe quantification and time-resolution of calcium during the ECC events. The Authors focused on calcium release unit structures at near-atomic resolution, the contribution of store-operated calcium entry (SOCE), and the role of mitochondria in calcium homeostasis during ECC. Evidently, those classical and new pharmacological tools and new experimental models such as induced pluripotent stem cells played a relevant role in the progress of the knowledge of the current status of the ECC.

A better understanding of the new phenomenon of calcium regulation during muscle contraction proposed as a calcium entry unit (CEU) was brought by Kittelberger et al. The physiology of CEU is defined by the presence of the two known proteins of the SOCE mechanism: the stromal interaction molecule-1 (STIM1) which acts as the calcium sensor in the sarcoplasmic reticulum (SR), and Orai1, a calcium-permeable channel in the T-tubule. Analyzing the structure of CEU in the sound poducing muscles of a teleost fish, evolved for extended activity at high-frequency during territorial and mating behavior. This novel mechanism was discovered to explain a rapid recovery of calcium, lost to the extracellular space during repetitive muscle contraction, and thus maintaining the SR calcium-load, necessary for high-frequency activity. The mechanism seems to rely on a re-arrangement of the SOCE proteins in the SR.

Another new function of SOCE to explain the role of calsequestrin-1 (casq-1) and its consequences on heat stroke was fully compiled by Protasi et al. Twenty years ago, the adventure to produce a knock-out mouse line for Casq-1 started, showing that the total absence of Casq-1 reduced the size of SR terminal cisternae on both soleus (slow-twitch muscle) and EDL (fast-twitch muscle), and remodeling the triads (SR-TT-SR), resulting in a reorganization of the CRU (DHPR and RyR-1), such as in the dyads observed in cardiomyocytes. This interesting structural adaptation in fast-twitch muscles is a consequence of the need for sufficient calcium release for muscle contraction, rather than the sole lack of Casq-1. Another remarkable finding was the mitochondrial damage visualized in Casq-1 null mice, which is a consequence of increased energy demand for calcium re-uptake and the mitochondriogenesis to support the excess of energy demand and calcium buffering capacity (reduced in the SR of Casq-1 null mice). Consequently, the long-term excess of cytosolic calcium increases the reactive oxygen species (ROS), a lethal effect on the muscle. The main physiological consequence of the Casq-1 KO-dependent anatomical variance was the reduction of the calcium transient and calcium SR-content, which altogether resulted in a reduction of force production. The phenotype of Casq-1 null mice was, however, undefined until the analysis of the dead male mice located in mating cages. After a series of studies involving short heat treatment, halothane, and hormonal treatment, the conclusion was that the Casq-1 null mice resemble the characteristics of acute heat shock syndrome, a disorder with no treatment. The final unsolved question of why Casq-1 null mice could survive as long as WT mice came along with the structural analysis of SOCE activation, through the assembly of CEUs. This was the compensatory mechanism used by Casq1-null mice to adjust for the lack of calcium in the SR.

Another particular and fine point of dysferlin-dependent calcium regulation at the triad was described by Lukyanenko et al. Dysferlin is a relevant structural protein located at the t-tubule, more precisely at the triad in skeletal muscle. The absence of dysferlin causes, for instance, Limb Girdle Type 2B (LGMD2B) and Miyoshi Myopathy (MMD1). In recent years and with the continuity of a project, Block and collaborators have found another interesting function of dysferlin, the one that can regulate calcium levels at the triad. In the A/J mouse cell culture model, the absence of dysferlin was evaluated before and after osmotic shock treatment causing an increase of basal calcium in sarcoplasm, reduced calcium transient, and therefore, dysregulation of calcium resulting in triggering the apoptosis response. In detail, after the osmotic shock, spontaneous calcium transients were followed by calcium sparks and waves, events associated with calcium-induced calcium release (CICR). This behavior was reversed by transfection of fibers to express dysferlin or by treatment with DHPR or RyR-1 blockers. Based on the evidence collected previously, the authors hypothesized that the absence of dysferlin inhibits the CICR upon muscle injury, buffering the excess of calcium. To test this hypothesis, the authors evaluated the calcium transient in an electrically stimulated KO-dysferlin A/J mouse model incubated with different calcium chelators (BAPTA, EGTA, Fluo-4) after being exposed to osmotic shock. Despite the differences among the chelators used in the study, the authors observed that the dysferlin-null phenotype was reversed with an extremely low concentration of the chelators in the sarcoplasm. The same evidence was observed when the authors transfected the muscle fibers with dysferlin containing GCaMP6f_u_ in place of its most N-terminal C2 domain. The advantage of the GCaMP6f_u_ is that it targets the triad junction like wild-type dysferlin. The results obtained suggested that the expression of dysferlin helped to re-establish calcium transient to normal levels and restored the calcium basal levels. Therefore, the authors demonstrated that dysferlin plays a relevant role in calcium homeostasis after muscle injury, which is crucial for the muscle repair processes after regular or sports activities.

Although the general aspects of the ECC have been described, there are still important open questions about the fine regulation during contraction, especially in pathological conditions. These new inquiries increased the complexity of the study model, such as new mutations and pharmacological tools. For instance, the functional role of nitric oxide (NO) during muscle contraction has been described with contradictions in some reports. The advanced technology allowed for improving the quality of the data, and thus described more precisely the role of NO and its source in skeletal muscle. A differential approach was proposed by Mosqueira et al., using a 3-D skeletal muscle tissue engineering technique. Forming muscle fibers from the C2C12 cell line, the authors further developed the technique, simplifying the cell culture and producing the engineered muscle fiber named myooids, permitting them to clarify the source of NO and its role. Validation of the myooids was done when related to diaphragm strips, showing comparable results for the expression of the most relevant ECC and NOS proteins, biophysical properties of force production, and the response to NO donor (SNAP), and unspecific NOS blocker (L-NAME). As predicted, the myooids only expressed two out of three NOS isoforms: the neuronal (nNOS) and inducible (iNOS). Another important advantage of 3-D tissue engineering using cell lines as a source for the muscle fiber is the high number of samples used in each experiment, thus having a solid statistical analysis and interpretation of the data. In total, the authors utilized 180 myooids to demonstrate that NO acts in two different points of ECC: one is dependent on the neuronal isoform of NOS, producing NO that negatively modulated the contraction force *via* soluble guanylyl cyclase (sGC)-protein kinase G (PKG) signaling pathway. This result supports previous results obtained from different skeletal muscles and, also relevant, provides another source of validation of the technique. Due to the facilitating protocol to produce the myooids, the authors also studied the iNOS-dependent NO source, finding a novel function of enhancing the excitability of the ECC in response to subthreshold electrical stimulation *via* two possible post-translational modifications: S-nitrosylation or nitration of tyrosine rings. This result might explain the adverse behavior found in different muscle pathologies where the iNOS is highly expressed. In summary, the authors developed a new approach to studying skeletal muscle function by producing muscle fibers from the C2C12 cell line, which can be genetically modified without the issues of lethality, time-consuming, and number of animals.

Together, these two editions of the Research Topic brought in a total of 18 novel and interesting manuscripts showing the current progress in the still exciting field of calcium function in the skeletal muscle.

